# The fission yeast FHIT homolog affects checkpoint control of proliferation and is regulated by mitochondrial electron transport

**DOI:** 10.1002/cbin.11241

**Published:** 2019-10-02

**Authors:** Johanna J. Sjölander, Per Sunnerhagen

**Affiliations:** ^1^ Department of Chemistry and Molecular Biology, Lundberg Laboratory University of Gothenburg P.O. Box 462 Göteborg SE‐405 30 Sweden

**Keywords:** 9‐1‐1 complex, Aph1, checkpoint proteins, hypoxia, *Schizosaccharomyces pombe*

## Abstract

Genetic analysis has strongly implicated human *FHIT* (Fragile Histidine Triad) as a tumor suppressor gene, being mutated in a large proportion of early‐stage cancers. The functions of the FHIT protein have, however, remained elusive. Here, we investigated *aph1* ^*+*^, the fission yeast homolog of *FHIT*, for functions related to checkpoint control and oxidative metabolism. In sublethal concentrations of DNA damaging agents, *aph1*Δ mutants grew with a substantially shorter lag phase. In *aph1*Δ mutants carrying a hypomorphic allele of *cds1* (the fission yeast homolog of Chk2), in addition, increased chromosome fragmentation and missegregation were found. We also found that under hypoxia or impaired electron transport function, the Aph1 protein level was strongly depressed. Previously, FHIT has been linked to regulation of the human 9‐1‐1 checkpoint complex constituted by Hus1, Rad1, and Rad9. In *Schizosaccharomyces pombe*, the levels of all three 9‐1‐1 proteins are all downregulated by hypoxia in similarity with Aph1. Moreover, deletion of the a*ph1* ^+^ gene reduced the Rad1 protein level, indicating a direct relationship between these two proteins. We conclude that the fission yeast FHIT homolog has a role in modulating DNA damage checkpoint function, possibly through an effect on the 9‐1‐1 complex, and that this effect may be critical under conditions of limiting oxidative metabolism and reoxygenation.

**Abbreviations**
Ap_3_A: diadenosine 5′,5‴‐P1,P3‐triphosphate
Ap_4_A: diadenosine 5′,5‴‐P1,P3‐tetraphosphate
DAPI: 4,6‐diamidino‐2‐phenylindole
DIC: differential interference contrast
Dox: doxorubicin
FHIT: fragile histidine triad
HU: hydroxyurea
PL: phleomycin
ROS: reactive oxygen species
YES: yeast extract with supplement

## Introduction

Inactivation of *FHIT*, through deletion, point mutation, or DNA methylation, is a very common event in cancer. It occurs in over half of human cancers, in particular in epithelial tumors, of which 70% suffer this impairment (Huebner and Croce, 2001; Saldivar et al., 2011). The first line of arguments for FHIT as a tumor suppressor protein was essentially based on this type of evidence. *FHIT* is located in the *FRA3B* locus, the most inducible fragile region in the human genome, revealing a cytologically distinguishable gap at chromosome 3p14.2 under certain experimental conditions (Durkin et al., 2008). Because of the location at a fragile site, it was initially questioned if FHIT was a true tumor suppressor or just frequently altered. *FHIT* ^+/−^ mice are, however, much more prone to develop tumors in response to carcinogen treatment (Fong et al., 2000; Zanesi et al., 2001), and both *FHIT* ^+/−^ and *FHIT* ^−/−^ mice have a higher frequency of spontaneous tumor development (Zanesi et al., 2001). The tumor development of FHIT deficient mice can also be partially repressed by *FHIT* gene therapy (Dumon et al., 2001) and re‐expression of FHIT in *fhit* deficient cells is able to induce apoptosis (Roz et al., 2002). The *FHIT* gene, or its expression, is commonly lost early in cancer development, and inactivation *of FHIT* is therefore proposed to result in a “mutator” phenotype (reviewed by Waters et al., 2014). Thus, the current view is that *FHIT*, due to its location on a fragile site, is prone to break in replication stress, and that its loss leads to more replication stress as well as to incompetence in appropriately handling new damage (Saldivar et al., 2012). This, in turn, leads to further progression of cancer development.

Though *FHIT* is now fully established as an important tumor suppressor, much less is understood about the actual cellular roles of the FHIT protein, in part because of the low abundance of the FHIT protein. FHIT was first described as a diadenosine 5′,5‴‐P1,P3‐triphosphate (Ap_3_A) hydrolase (Murphy et al., 2000). Expression of wt FHIT or a FHIT^*H96N*^ mutant protein, lacking the Ap_3_A hydrolase activity, were however equally effective in abrogating tumor progression (Siprashvili et al., 1997), indicating that it is rather the substrate binding, not cleavage, that is important for the anti‐tumor activities. More recently, in vitro and in vivo studies in *Saccharomyces cerevisiae,* showed that the budding yeast homolog of FHIT, Hnt2 as well as human FHIT catabolize m^7^GpppG dinucleotides generated from the 5′‐cap structures from degraded messenger RNAs (mRNAs) (Taverniti and Séraphin, 2015). Inefficient degradation of m^7^GpppG results in elevated concentrations of this intermediate, which has been reported to inhibit mRNA splicing (Izaurralde et al., 1994) and export to the cytoplasm of nuclear RNAs (Hamm and Mattaj, 1990) as well as to promote mRNA deadenylation (Wu et al., 2009).

The FHIT protein is located in the cytoplasm and nucleus (Zhao et al., 2006) as well as in mitochondria (Druck, et al., 2019). In mitochondria, FHIT physically interacts with ferredoxin reductase, and this interaction is important for induction of apoptosis through elevated production of reactive oxygen species (ROS) (Druck et al., 2019). FHIT has also been implicated in regulation of checkpoint responses. In *fhit* ^*−/−*^ cells, Chk1 is constitutively hyperactive, resulting in enhanced S and G2 checkpoint responses (Hu et al., 2005). Loss of FHIT also confers lower levels of hHus1 (Ishii et al., 2006), a protein in the 9‐1‐1 DNA sliding clamp complex involved in DNA damage sensing, DNA repair, and induction of checkpoint control (reviewed by Parrilla‐Castellar et al., 2004).

Both fission and budding yeast FHIT orthologs exist. *Schizosaccharomyces pombe* Aph1 has 43% amino acid sequence identity and 55% similarity with human FHIT (Huang et al., 1995), and 41% identity and 57% similarity with Hnt2 of the budding yeast *S. cerevisiae* (Chen et al., 1998). Only limited functional studies have been performed with the yeast orthologs, mostly inspired by the dinucleotide hydrolase activity of the enzymes. For both yeast orthologs, gene disruption leads to strong accumulation of intracellular diadenosine oligophosphate (Ingram and Barnes, 2000; Rubio‐Texeira et al., 2002), and overexpression of *aph1* ^*+*^ lowers these concentrations, as expected (Ingram and Barnes, 2000). Studies of *S. cerevisiae* Hnt2 have already shed some light on FHIT function, with the binding and cleavage of the m^7^GpppG cap structures (Taverniti and Séraphin, 2015). In a previous genetic interaction screen (Ryan et al., 2012), Aph1 was found to interact positively with the mitochondrial TOM complex (translocase of the outer membrane) as well as Hus1 in the 9‐1‐1 checkpoint complex. Studying the FHIT orthologs in budding and fission yeast is a promising starting point for elucidating its molecular functions.

In this study, we wanted to address the function of FHIT through its ortholog Aph1 in the genetically amenable fission yeast. We started by investigating effects on cellular proliferation by a deletion of *aph1* ^*+*^, and found that loss of Aph1 leads to unregulated proliferation of cells exposed to a number of DNA‐damaging agents. Furthermore, the combination of *aph1∆* and a partial defect in the checkpoint protein Cds1 (ortholog of human Chk2) results in elevated chromosome fragmentation and missegregation in doxorubicin (Dox).

The human FHIT interaction with the electron transport chain (Druck et al., 2019) and the Aph1 interaction with the TOM complex (Ryan et al., 2012) inspired us to also investigate a possible dependence of Aph1 levels on oxidative phosphorylation. We found that Aph1 protein levels were strongly downregulated in hypoxia or low glucose levels as well as by blocking mitochondrial electron transport.

As FHIT has been shown to modulate hHus1 expression (Ishii et al., 2006), we investigated a possible link between Aph1 and the 9‐1‐1 proteins. Aph1 loss additionally resulted in a strong reduction of the Rad1 protein level, indicating a positive regulation of the 9‐1‐1 complex. Interestingly all three 9‐1‐1 proteins; Rad1, Hus1, and Rad9, were similarly downregulated in hypoxia.

Altogether it seems that some features are conserved between human FHIT and fission yeast Aph1. Mutation of those genes resulted in unregulated proliferation and DNA damage. That Aph1, in contrast to FHIT (Murphy et al., 2000), prefers to cleave Ap_4_A over Ap_3_A (Ingram and Barnes, 2000), is apparently not important for these conserved functions. Therefore, *S. pombe* should be an attractive model for studies on the anti‐proliferative functions of Aph1/FHIT at the cellular level.

## Materials and methods

### *S. pombe* strains and growth conditions

All strains are listed in Table 1. Growth was at 30°C in YES medium, unless indicated otherwise.

**Table 1:** *S. pombe* strains used in this study

Strain	Genotype	Source or reference
972*h* ^−^	*h* ^−^	Lab stock
JJS30	*h* ^−^ *aph1::KanMX*	This work
JJS31	*h* ^−^ *aph1* ^*+*^:*(HA)*_*3*_:*HphMX*	This work
JJS32	*h* ^−^ *rad1* ^*+*^:*(HA)*_*3*_:*HphMX*	This work
JJS33	*h* ^−^ *rad1* ^*+*^:*(HA)*_*3*_:*HphMX aph1::KanMX*	This work
JJS34	*h* ^−^ *rad1* ^*+*^:*(HA)*_*3*_:*HphMX hus1::NatMX*	This work
JJS35	*h* ^−^ *rad1* ^*+*^:*(HA)*_*3*_:*HphMX rad9::NatMX*	This work
JJS36	*h* ^−^ *hus1* ^*+*^:*(HA)*_*3*_:*HphMX*	This work
JJS46	*h* ^−^ *hus1* ^*+*^:*(HA)*_*3*_:* HphMX aph1::KanMX*	This work
JJS37	*h* ^−^ *hus1* ^*+*^:*(HA)*_*3*_:*HphMX rad1::NatMX*	This work
JJS38	*h* ^−^ *hus1* ^*+*^:*(HA)*_*3*_:*HphMX rad9::NatMX*	This work
JJS39	*h* ^−^ *rad9* ^*+*^:*(HA)*_*3*_:*HphMX*	This work
JJS40	*h* ^−^ *rad9* ^*+*^:*(HA)*_*3*_:*HphMX aph1::KanMX*	This work
JJS41	*h* ^−^ *rad9* ^*+*^:*(HA)*_*3*_:*HphMX rad1::NatMX*	This work
JJS42	*h* ^−^ *rad9* ^*+*^:*(HA)*_*3*_:*HphMX hus1::NatMX*	This work
NW222	*h* ^−^ *ade6‐216 leu1‐32 chk1* ^*+*^:*(HA)*_*3*_	N. Walworth
JJS43	*h* ^−^ *ade6‐216 leu1‐32 chk1* ^*+*^:*(HA)*_*3*_ *cds1Δ::KanMX*	This work
JJS44	*h* ^−^ *ade6‐216 leu1‐32 chk1* ^*+*^:*(HA)*_*3*_ *cds1:(myc)*_*9*_:*HphMX*	This work
JJS45	*h* ^−^ *ade6‐216 leu1‐32 chk1* ^*+*^:*(HA)*_*3*_ *cds1:(myc)*_*9*_:*HphMX aph1::KanMX*	This work

### Quantification of growth rate and lag phase

Pre‐cultures were grown to saturation density by inoculation in 10 mL YES and growth for 48 h. Stationary phase cultures were thereafter transferred to microtiter plates containing YES with the indicated additions to a final OD_600nm_ of 0.125 in 200 μL total volume per well containing either 9 mM hydroxyurea (HU), 0.2 μM phleomycin (PL), or 19 μg/mL Dox. YES alone was used for controls.

Air‐permeable film (Breathe‐Easy, Diversified Biotech, Boston, USA) was used instead of a lid, as differential oxygenation depending on the position of the plate affected results in the growth experiments. Cell growth with high‐intensity shaking was monitored every 20 min. in a Bioscreen Analyzer C (Growth Curves USA) for 72 h as described (Warringer and Blomberg, 2003). Raw data were processed with the PRECOG tool (Fernandez‐Ricaud et al., 2016).

### HU and UV survival tests

Logarithmically growing cells (OD_600nm_ ≈ 0.5) were diluted to OD_600nm_ = 0.3, and serially diluted 1:3 in a 96 well plate. From each well, 5 μL was plated onto YES agar with or without 5 mM HU. Irradiation with UV at 254 nm was 200 µJ/cm^2^.

### Microscopy

Evaluation of chromosome fragmentation/missegregation: cells were fixed with ethanol and stained with 4′,6‐diamidino‐2‐phenylindole (DAPI) essentially, as described (Alao et al., 2014). Images were obtained with a Zeiss AxioCam on a Zeiss Axioplan 2 microscope with a ×100 objective, using the appropriate filter (DAPI or DIC). For quantifications at least 200 cells/replicate was counted. Three independent experiments were quantified.

Survival assay with propidium iodide (PI): Live cells were, at the indicated time point, stained with 10 µg/mL PI and subjected tor analysis by microscopy. Images were obtained with a Zeiss AxioCam on a Zeiss Axioplan 2 microscope with a ×100 objective, using the appropriate filter (red fluorescence or DIC). For quantifications, at least 200 cells/replicate were counted. Three independent experiments were quantified.

### Growth in hypoxia

Hypoxic conditions were generated by continuously leading nitrogen gas into the liquid medium. Nitrogen gas was first led through a tube down into distilled water, to moisten the gas, and then further into a closed chamber with multiple tubing, equally distributing the gas into each culture, with the same total volume, within the same experiment.

### Western blot

Cell pellets were collected by centrifugation and snap‐frozen on dry ice. Cells were thawed on ice and lysed by shaking with acid‐washed glass beads in a FastPrep FP120 device (Savant) at speed 5 for 30 s. Lysis was performed in lysis buffer A (50 mM NaCl, 50 mM Tris pH 7.6, 0.2% Triton X‐100, 0.25% NP40) containing phosphatase inhibitor cocktail 04906837001 and protease inhibitor cocktail 04693159001 (Roche). Protein concentration was determined using the BCA assay. Equal protein concentrations of each sample were loaded and proteins were separated on a sodium dodecyl sulfate‐polyacrylamide gel electrophoresis (SDS‐PAGE) and blotted onto nitrocellulose membranes.

HA epitope‐tagged versions of Aph1, Rad1, Hus1‐, and Rad9 as well as Chk1 were detected by mouse anti‐HA Sc7392 (Santa Cruz Biotechnology) or mouse anti‐HA 2367 S from Cell Signaling Technology (Bionordika AB, Stockholm, Sweden). Cds1 was detected using Mouse anti‐c‐myc Sc40 (Santa Cruz Biotechnology). To be able to detect the low‐abundance Aph1 protein, incubation with the primary antibody was for 72 h. The loading control was α‐tubulin detected by mouse anti‐α‐tubulin T5168 (Sigma), or staining of total protein with Ponceau S Solution (Sigma). The secondary antibody was horseradish peroxidase‐coupled α‐mouse A4416 (Sigma).

## Results

### Deletion of *aph1* ^*+*^ leads to proliferation in sublethal concentrations of genotoxins

**Figure 1:**
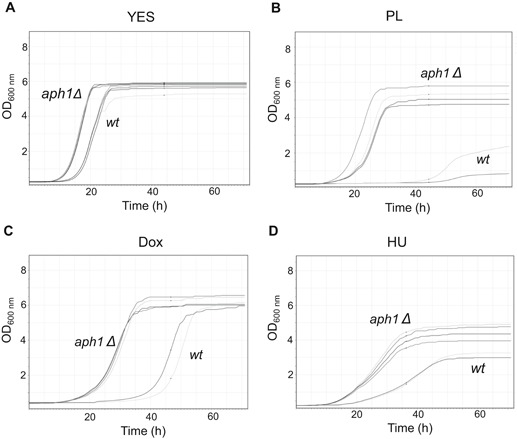
**Loss of Aph1 leads to unregulated proliferation in sublethal concentrations of genotoxic agents.** Stationary phase cultures of wt (972 *h* ^−^) and *aph1Δ* (JJS30), 48 h from inoculation, were re‐diluted in fresh media containing YES with or without genotoxins for re‐entry of growth for 72 h. Multiple individual curves from independent precultures are shown from the Bioscreen C analyzer. Treatments were (A) YES alone; (B) YES containing 0.2 μM PL; (C) YES with 19 μg/mL Dox; (D) YES with 9.5 mM HU.

The starting point in this assay was a stationary phase culture (48 h from inoculation), diluted into fresh YES media for reinitiating of growth. We used a Bioscreen C analyzer (Growth curves USA) to generate multiple independent growth curves simultaneously. *aph1Δ* cells always re‐entered growth slightly earlier than wt in control conditions (Figure 1A). In the presence of the genotoxic compounds PL (Figure 1B), Dox (Figure 1C) or HU (Figure 1D), *aph1Δ* cells displayed a much shorter lag, and also reached a higher cell density compared to wt 972*h* ^−^ cells in PL and HU (Figures 1B and 1D). Thus, *aph1Δ* mutants are clearly more prone to restart growth under genotoxic conditions, showing that proliferation control is deregulated in this mutant.

### Aph1 is needed for adaptation to stationary phase

We investigated survival in a density saturated culture. We inoculated cells in liquid YES media to an OD_600_ of 0.3, and thereafter evaluated survival by a propidium iodide (PI) permeability/exclusion assay, where intact cells exclude PI (Moreno et al., 1991). After 24 h, where wt and *aph1Δ* were approximately reaching maximum density, survival was indistinguishable between wt and *aph1Δ*, whereas after 72 h, more *aph1Δ* cells were PI permeable (Figure S1A), indicating lower survival in the prolonged stationary phase in cells lacking Aph1. This implies that *aph1Δ* has a problem adapting to a non‐proliferation mode, which may explain why *aph1Δ* mutants reinitiate growth earlier also in the control conditions in the Bioscreen (Figure 1A).

We also followed the expression of Aph1 protein during growth from the early logarithmic phase to the stationary phase. The Aph1 protein level was rising throughout the course (up to OD_600nm_ = 2.5), but in the stationary phase (OD_600nm_ = 9.9), Aph1 protein was undetectable (Figure S1B). Thus, Aph1 levels build up during proliferation and sharply decline when cells cease growth. One interpretation of the above findings is that a graded expression during proliferation and non‐proliferation aids to appropriately adapt between growth and non‐growth.

### Aph1 influences checkpoint control

**Figure 2:**
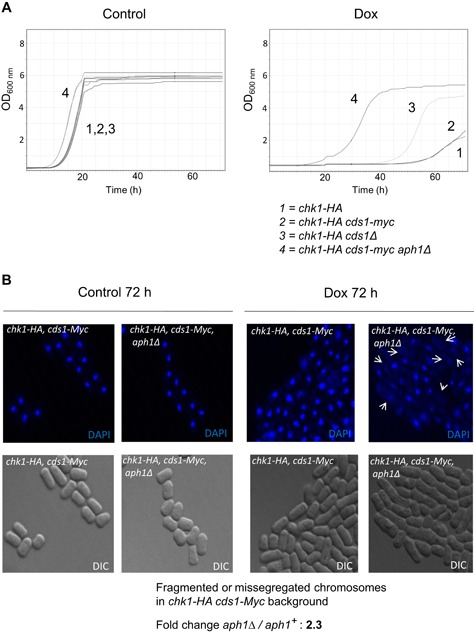
**Loss of Aph1 leads to checkpoint control override in cells carrying a *cds1* hypomorphic allele leading to elevated chromosome fragmentation in doxorubicin.** The *aph1∆* allele in a *chk1‐HA cds1‐(myc)_9_* background leads to a shorter lag phase and growth to higher densities. Stationary phase cultures of *chk1‐HA* (NW222), *chk1‐HA cds1‐(myc)_9_* (JJS44), *chk1‐HA cds1Δ* (JJS43), and *chk1‐HA cds1‐(myc)_9_ aph1Δ* (JJS45), 48 h from inoculation, were re‐diluted in fresh media containing YES with or without Dox for re‐entry of growth 72 h in a Bioscreen C analyzer. (A) Representative growth curves. Treatments were YES only or 19 μM Dox. (B) The higher proliferation in *chk1‐HA cds1‐(myc)_9_ aph1Δ* (JJS45) was accompanied by elevated chromosome fragmentation and/or missegregation. Cells from *chk1‐HA cds1‐(myc)_9_* (JJS44) *chk1‐HA cds1‐(myc)_9_ aph1Δ* (JJS45) were fixed in ethanol after 72 h of growth, stained with 4′,6‐diamidino‐2‐phenylindole (DAPI) and evaluated by microscopy for fragmentation and missegregation. Cells from three independent Bioscreen experiments were collected by centrifugation, cleared of growth media and vortexed in 70% ethanol for 30 s. This was done directly at the end of the 72 h Bioscreen run. Representative pictures of DAPI‐stained cells are shown. Note that although there is some missegregation present in Dox‐treated *chk1‐HA cds1‐(myc)_9_ wt* (JJS44), this phenotype is much stronger in *cds1‐(myc)9 aph1Δ* (JJS45) where some cells (white arrows) appear to lack nuclei entirely. For quantification of cells with fragmented and/or cut chromosomes, at least 200 cells were counted for each of the three replicates.

As FHIT function has been shown to modulate Chk1 and Chk2 activation (Ishii et al., 2006; Yutori et al., 2008), two important kinases involved in checkpoint control, we introduced a (Myc)_9_ epitope tag to the C‐terminal of Cds1. This was done with the purpose of following the Cds1 protein band shift upon activation through phosphorylation by Rad3 (Lindsay et al., 1998) in a genetic background that already had a HA tag on Chk1, for following Chk1 activation that also results in a band shift (Walworth and Bernards, 1996). Although we did observe the expected band shift upon Cds1 activation (Figure S2A), unexpectedly, the introduction of the Myc_(9)_ tag partially interfered with Cds1 function (Figure S2B). Activation of Chk1 by HU was seen in the double‐tagged strain, indicating that DNA damage activated Chk1 to compensate for the loss of Cds1. Chk1 is normally not activated by HU, but is known to be activated by HU in *cds1Δ* (Lindsay et al., 1998). This was paralleled by the survival on 5 mM HU plates, where the sensitivity of this *chk1‐HA cds1‐(myc)*_*9*_ strain was intermediate between the *chk1‐HA* strain and the *chk1‐HA cds1Δ strain* (Figure S2B). Deletion *of aph1* ^*+*^ in a *chk1‐HA cds1‐(myc)*_*9*_ background did suppress a growth delay in HU (Figure S2D) and Dox (Figure 2A). However, *aph1*∆ in this background gave no clear additional phenotype on survival in HU (Figure S2D). When inspecting cells in the microscope after 72 h growth in 19 µg/mL Dox, the frequency of cells with cut and/or fragmented chromosomes was clearly higher in *aph1Δ* than in wt cells (Figure 2B). Among cells with cut/fragmented chromosomes, the defect was more pronounced in *aph1Δ* with some cells having no nuclei staining but only punctuate cytoplasmic staining with DAPI (Figure 2B, see white arrows in DAPI picture of *chk1‐HA cds1‐(myc)*_*9*_ *aph1Δ*).

Even though the *aph1*∆ knockout in the strain carrying the partially defective *cds1‐(myc)*_*9*_ allele resulted in an increased frequency of fragmented/cut chromosomes in the presence of genotoxins, proliferation thus continued unabated, indicating loss of cell cycle checkpoint control. It is therefore plausible that the Aph1 is involved in checkpoint control of proliferation.

### Aph1 protein expression is suppressed by hypoxia

**Figure 3:**
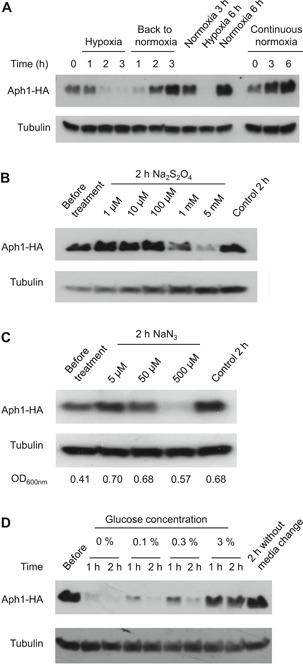
**Aph1 is downregulated in conditions of low mitochondrial electron transport.** Logarithmically growing *aph1‐HA* (JJS31) cells were subjected to different treatments and analyzed by western blotting. (A and B) Hypoxia results in a reversible decrease in the Aph1 protein level. (A) Cells were subjected to either hypoxia induced by leading N_2_ into the culture or normoxia. (B) Cells were subjected to hypoxia introduced by dithionite of the indicated concentration for 2 h. (C) Blocking of mitochondrial electron transport by addition of sodium azide leads to a reduction in Aph1 protein level. Cells were treated with NaN_3_ of the indicated concentration for 2 h. (D) Reducing the glucose level in media also lowers the Aph1 protein level. Cells were subjected to a media change where the new medium contained different glucose concentrations.

Knowing that FHIT is partially localized in mitochondria (Druck et al., 2019), and that the FHIT‐controlled microRNA miR‐30c is suppressed under hypoxic conditions (Huang et al., 2013), we speculated that Aph1/FHIT expression may be influenced by the oxygenation status of the cells. We investigated Aph1 levels in cells growing in hypoxia, as described in the Materials and Methods section. As seen in Figure 3A, after 2 h in hypoxic conditions, the Aph1 protein level is strongly reduced, and after 3 h, Aph1 is virtually absent. This process is reversible, as restoring normoxia brings Aph1 back to near‐normal levels within 2 h (Figure 3A). To ascertain whether this effect was due to the oxygenation status, we used a different method to produce hypoxia, the reducing agent sodium dithionite. At higher concentrations, upwards of 1 mM, exposure to dithionite for 2 h had the same effect on Aph1 as displacing air with nitrogen gas (Figure 3B). To examine whether these effects on Aph1 were due to decreased respiratory activity, we used the electron transport chain poison azide. As seen in Figure 3C, treating cells for 2 h with sodium azide also caused Aph1 protein to decrease. Importantly, even at the highest azide concentration (0.5 mM), the optical density of the culture was increasing, demonstrating that the cells were still alive. This indicates that it is not the redox state of the environment *per se* that causes the reduction of Aph1, but rather the activity of the mitochondrial electron transport chain. To further investigate the relationship between the Aph1 level and energy metabolism rate, we reduced the glucose concentration in the medium for exponentially growing cells. As seen in Figure 3D, a reduction of glucose concentration from 3% to 0.3% caused a marked reduction of Aph1 within 1 h, and after 2 h the Aph1 level was even lower. Reducing glucose to 0.1% or eliminating it altogether results in further drops of Aph1 protein levels. Together, these observations establish a strong correlation between electron transport chain activity and the level of Aph1 protein.

### Expression of Rad1 is dependent on Aph1

**Figure 4:**
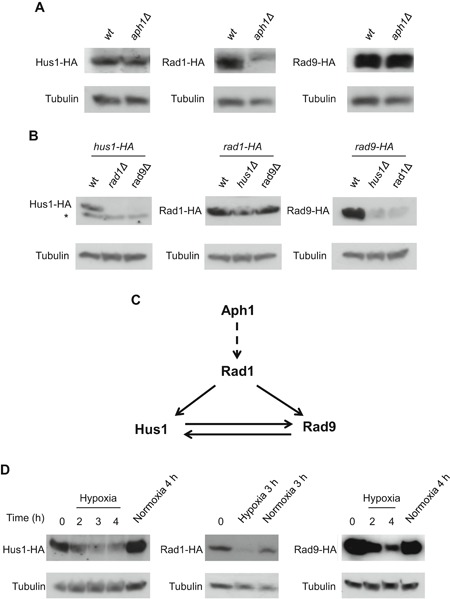
**Aph1 regulates the level of Rad1, and all 9‐1‐1 proteins are downregulated in hypoxia**. Logarithmic growing cells expressing C‐terminally (HA)_3_ tagged versions of Hus1, Rad1, and Rad9 from their endogenous promoters were subjected to different treatments or investigated in different genetic backgrounds, and analyzed by western blotting. (A) Rad1, but not Hus1 or Rad9 protein levels, is reduced by deleting *aph1^+^*. Strains used were *hus1‐HA* (JJS36), *hus1‐HA aph1Δ* (JJS46), *rad1‐HA* (JJS32), *rad1‐HA aph1Δ* (JJS33), *rad9‐HA* (JJS39), *rad9‐HA aph1Δ* (JJS40). (B) Hus1 and Rad9, but not Rad1 protein levels are reduced by the absence of one other 9‐1‐1 complex member. Strains used were *hus1‐HA* (JJS36), *hus1‐HA rad1Δ* (JJS37), *hus1‐HA rad9Δ* (JJS38), *rad1‐HA* (JJS32), *rad1‐HA hus1Δ* (JJS34), *rad1‐HA rad9Δ* (JJS35), *rad9‐HA* (JJS39), *rad9‐HA hus1Δ* (JJS42), *rad9‐HA rad1Δ* (JJS41). (C) Model based on the results from A and B, showing Aph1 regulation of the 9‐1‐1 complex through the Rad1 protein level. (D) The protein levels of Hus1, Rad1, and Rad9 are like Aph1 all down‐regulated in hypoxia. Hypoxia was induced by leading N_2_ gas into cultures of cells from strains *hus1‐HA* (JJS36), *rad1‐HA* (JJS32) and *rad9‐HA* (JJS39). *marks a non‐specific background band detected by the anti‐HA antibody.

In logarithmically growing unstressed *aph1∆* cells, the Rad1 level was strongly reduced compared to wt. The expression of Rad9 and Hus1 was virtually unaltered in *aph1∆* mutants, however (Figure 4A).

In *rad1∆* and *rad9∆* mutants, the level of Hus1 is markedly suppressed; likewise, Rad9 levels are suppressed in *rad1∆* and *hus1∆* mutants (Figure 4B). By contrast, deletion of *rad9* ^*+*^ or *hus1* ^*+*^ does not appreciably affect the expression of Rad1. Thus, Rad1 influences expression of the other two 9‐1‐1 proteins, but not vice versa; this pattern is analogous to what has been observed for the human 9‐1‐1 orthologs (Bao et al., 2004).

Considering these findings in combination with our data showing lower levels of Rad9 and Hus1 in *rad1∆* mutants (Figure 4A), it appears that Aph1 has a more direct influence on Rad1 levels than on the other 9‐1‐1 proteins (Figure 4C). Furthermore, while eliminating Rad1 altogether (as in *rad1∆* mutants, Figure 4B) does suppress Rad9 and Hus1 levels, reducing Rad1 partially (as in *aph1∆* mutants, Figure 4A) is not enough to achieve this effect.

### The 9‐1‐1 proteins are, like Aph1, downregulated in hypoxia

Given the effects on the Rad1 level of deleting *aph1* ^*+*^, the effect of deleting *rad1* ^*+*^ on the levels of other 9‐1‐1 proteins, and our finding that hypoxia causes depletion of Aph1 protein; it is natural to ask whether hypoxia also affects the levels of the 9‐1‐1 proteins. As shown in Figure 4D, there was indeed a clear drop in the levels of all three 9‐1‐1 proteins within 2–3 h after purging oxygen from the medium.

## Discussion

We have shown that *aph1Δ* mutants restart growth from the stationary phase at a much earlier point when exposed to various genotoxic agents (PL, HU, and Dox) than wt (Figures 1B–D), indicating that elimination of Aph1 results in unresponsiveness to checkpoint control. The *aph1Δ* mutants also reentered growth slightly earlier even in normal conditions (Figure 1A), indicating that deleting *aph1* ^*+*^ results in a phenotype also generally more prone to restart growth from a non‐proliferating state. We further noted that *aph1Δ* cells are defective in adapting to the stationary phase, as seen by the defective survival in the prolonged stationary phase (Figure S1). Thus, the rapid re‐entry into proliferation from the stationary phase may represent the fact that mutant cells are still partially set for proliferation, from the previous logarithmic phase.

In the case of combining a partially defective *cds1‐Myc* allele with *aph1Δ* (Figure S2), the shorter lag and higher yield seen in Dox (Figure 2A) was also accompanied by a higher fraction of fragmented and/or cut chromosomes in Dox compared to that in the partially defective *cds1‐Myc* allele alone (Figure 2B). These observations, together, point towards a role for Aph1 in cell cycle checkpoint control under genotoxic conditions.

Human FHIT has been shown to be located in the cytosol, nucleus (Zhao et al., 2006) and mitochondria (Druck et al., 2019). Aph1 is located in the cytosol and nucleus (Matsuyama et al., 2006), and is predicted by its similarity to the budding yeast Hnt2, but not actually shown, to also be present in mitochondria. We have not investigated the localization of Aph1 in fission yeast, however, its strong dependence on oxygenation (Figures 3A and 3B), and on a functioning mitochondrial electron transport chain (Figure 3C) are indications of an Aph1 pool within the mitochondria. The human FHIT mitochondrial pool physically interacts with ferredoxin reductase, and this interaction has been proposed to confer a higher ROS production leading to induction of apoptosis (Druck et al., 2019). In the unicellular organism *S. pombe*, there are indications of programmed cell death with characteristics reminiscent of apoptosis (Su et al., 2017). We have not investigated if there is a link between Aph1 and programmed cell death in *S. pombe*; however, our observations with the dysregulated restart of proliferation in sublethal concentrations of DNA‐damaging agents (Figures 1 and 2, Figure S2C), and failure of efficient adaptation to non‐growth upon nutrient limitation (Figure S1), rather point towards a role for Aph1 in control of proliferation. We, therefore, propose that Aph1 links aerobic respiration in mitochondria with proliferation control under genotoxic conditions as well as in nutrient limitation. The earlier observation that overexpression of Aph1 leads to longer generation time (Ingram and Barnes, 2000) is in line with the anti‐proliferative functions for *S. pombe* Aph1.

Human FHIT preferentially hydrolyzes Ap_3_A over Ap_4_A (Murphy et al., 2000). As a FHIT^*H96N*^ mutant protein binds Ap_3_A efficiently, but has a strong catalytic defect, and nevertheless maintains the anti‐tumor capabilities of FHIT (Siprashvili et al., 1997), the leading hypothesis has been that FHIT bound to its substrate constitutes the active tumor suppressor signaling unit. In contrast to human FHIT, *S. pombe* Aph1 prefers to hydrolyze Ap_4_A over Ap_3_A (Ingram and Barnes, 2000). Despite this difference, *aph1Δ* mutants display unrestrained restart of growth in genotoxic conditions (Figures 1 and 2), indicating that this difference is not important for the anti‐proliferative functions of Aph1.

FHIT has been shown to regulate both the levels (Kiss et al., 2018) and the translation of mRNAs important in cancer development (Kiss et al., 2017a). Thus, it is plausible that some of the biochemical basis for the tumor suppressor functions of FHIT is through its actions on mRNAs (Kiss et al., 2017b). As the binding rather than hydrolysis of the substrate seems to be important for the anti‐tumor functions of FHIT, one can also not exclude the fact that other substrates similar in structures to Ap_3_A and m^7^GpppG may be important for FHIT cellular functions. In human cells, it was recently shown that FHIT positively regulates TK1 (Thymidine kinase 1) by stimulation of translation of *TK1* transcripts (Kiss, Waters et al., 2017b) as well as a number of other important tumor suppressor transcripts (Kiss et al., 2017a). The reduction of TK1 leads to replication stress and the DNA strand breaks because of a reduction in the dTTP pool (Saldivar et al., 2012). The stimulation of TK1 translation is interestingly linked to the binding of FHIT to m^7^GpppN cap structures (Kiss et al., 2017b) rather than to the substrate Ap_3_A. Importantly the FHIT^*H96N*^ mutant protein, which has no catalytic activity against Ap_3_A nor to m^7^GpppG (Taverniti and Séraphin, 2015), is as effective in stimulation of translation as the wt version. Similarly wt FHIT and the FHIT^*H96N*^ mutant proteins are equally effective in preventing DNA damage, whereas another mutant allele, FHIT^*Y114F*^, defective in m^7^GpppG binding, was defective in this function (Kiss et al., 2017b). Therefore, the translational impact on *TK1* may be through binding to 5′ cap structures either generated by 3′–5′ mRNA decay, that may otherwise compete for eIF4E binding as proposed (Kiss et al., 2017b), or to the 5′ cap while still on intact mRNA. As we have shown that Aph1 positively regulates Rad1 in the 9‐1‐1 complex (Figure 4A), it would be interesting to see if this occurs through binding to 5′ cap structures, as for FHIT in the regulation of TK1.

Deleting *aph1* ^*+*^ resulted in a strong reduction of the Rad1 protein level (Figure 4A), confirming a link between Aph1 and the 9‐1‐1 complex. As the 9‐1‐1 complex is a heterotrimer (Dore et al., 2009), this should result in a lower pool of this important complex. This parallels the finding that suppression of human FHIT levels leads to downregulation of the 9‐1‐1 protein hHus1 (Ishii et al., 2006). Deletion of Rad1 resulted in downregulation of both Rad9 and Hus1 protein levels, whereas deletion of Rad9 and Hus1 did not significantly affect the Rad1 level (Figure 4B). The link between Aph1 and the 9‐1‐1 complex seems to be through Rad1, as *aph1Δ* mutants showed a reduced Rad1 protein level, however, not those of the other two 9‐1‐1 constituents (Figure 4B). Thus, the reduction in Rad1 protein level seen in *aph1Δ* did not reduce Hus1 or Rad9 levels, indicating that the low Rad1 level still present in *aph1Δ* is enough to sustain Hus1 and Rad9 levels. Interestingly all three 9‐1‐1 proteins were downregulated in hypoxia (Figure 4D) similar to Aph1 (Figures 3A and 3B), indicating that cells reentering to normal oxygenation from hypoxia may have to face the higher oxygen level with a lagging 9‐1‐1 complex level, potentially resulting in DNA damage.

We have characterized some aspects of the regulation of Aph1 protein level in fission yeast with respect to growth conditions such as the growth phase, oxygenation, and glucose availability (Figure 3 and Figure S1B). The downregulation of the *S. pombe* Aph1 protein level in hypoxia is reversed when regaining normoxia (Figure 3A). It is unknown if human FHIT is also regulated by oxygen availability and activity of electron transport. FHIT is however downregulated in human pulmonary arterial hypertension (PAH), and FHIT positively regulates BMPR2 (bone morphogenetic protein receptor type 2), also downregulated in PAH, and important for the development of PAH (Dannewitz Prosseda et al., 2019). Pulmonary hypertension (PH) can be caused by hypoxia (Weitzenblum and Chaouat, 2001) or conditions mimicking hypoxia by high NO production leading to inhibition of mitochondrial electron transport and induction of Hif1α (hypoxia‐inducible factor α) (Fijalkowska et al., 2010). An emerging “metabolic theory of PAH” states that also PAH may be caused by mitochondria‐based metabolic abnormalities resulting in reduced oxidative phosphorylation (Paulin and Michelakis, 2014). This indicates that FHIT may also be downregulated in hypoxia, or in other conditions leading to blockage of mitochondrial electron transport. We, therefore, speculate that FHIT/Aph1 downregulation in lower oxygen and upregulation when oxygen returns may both be important in hypoxia and reoxygenation. In agreement with this, FHIT^−/−^ mice had more severe pulmonary hypertension and this was not reversed in normoxia, as opposed to in FHIT^+/+^ mice (Dannewitz Prosseda et al., 2019). If FHIT is important for the appropriate cellular responses to hypoxia/reoxygenation, loss of these functions would be important also in cancer development, where hypoxia is a common feature even in small tumors (Li and O'Donoghue, 2008), and where reoxygenation of hypoxic cancer cells from tumors results in a high colonization ability (Young and Hill, 1990). Mammalian FHIT also positively regulates the miR‐30c microRNA. Through this action on miR‐30c, FHIT counteracts the epithelial–mesenchymal transition (EMT) in human lung cancer cells (Suh et al., 2014). Hypoxia induces downregulation of miR‐30c (Huang et al., 2013), and this downregulation promotes EMT in human carcinoma cells. It is thus a credible notion that the suppression of Aph1 expression in hypoxia that we have observed similarly affects the expression of other gene products, as the Aph1 reduction in hypoxia (Figure 3A) is correlated with strongly reduced protein levels of all three 9‐1‐1 proteins (Figure 4D).

### Conclusions

Historically, fission yeast has been an important starting point for elucidating basic cell cycle and checkpoint mechanisms. Altogether, it seems that some aspects are conserved between fission yeast Aph1 and human FHIT, including anti‐proliferative functions, regulation of the 9‐1‐1 complex, and a likely association with the mitochondrial electron transport chain. Aph1 in fission yeast, with its extensive possibilities for genetic analysis, should, therefore, be an excellent model to elucidate the biological role of the human FHIT protein.

## Acknowledgments and funding

This work was supported by grants from the Royal Society of Arts and Sciences, Sigurd and Elsa Goljes Minne and the Lennander's Foundations to J.J.S., and from the Swedish Cancer Fund (2013‐512 and 2016‐378) to P.S.

## Supporting information


**Figure S1. Aph1 is needed for adaptation to stationary phase.A,B)** *aph1Δ* mutants are sensitive to prolonged stationary phase. **A)** OD_600 nm_ was measured at 24 and 72 h from inoculation. The growth curves show that both wt (972 h‐) and *aph1Δ* (JJS30) reach the same maximal density at 24 h. **B)** At 24 h, wt (972 *h* ^‐^) and *aph1Δ* (JJS30) cells are equally effective at excluding PI, whereas at 72 h *aph1Δ* mutants have more PI permeable cells, indicating that these cells are no longer viable.

**Figure S2. *cds1‐(myc)_9_* is a hypomorphic allele, and *aph1Δ* in this background results in more proliferation than wt under exposure to DNA damaging agents. A)** When activated by HU, Cds1 C‐terminally tagged with (Myc)_9_ migrates slower as expected, and the *aph1Δ* allele does not change this. *chk1‐HA* (NW222), *chk1‐HA cds1‐(myc)_9_* (JJS44) and *chk1‐HA cds1‐(myc)_9_ aph1Δ* (JJS45) were treated for 2 h with 20 mM HU, and activation of Cds1 was investigated through Western blotting by presence of a band shift to a slower migrating band. **B)** The compromised Cds1 function caused by the (Myc)_9_ tag results in Chk1 activation in HU, indicating DNA damage. Strains *chk1‐HA* (NW222), *chk1‐HA cds1Δ* (JJS43), *chk1‐HA cds1‐(myc)_9_* (JJS44), and *chk1‐HA cds1‐(myc)_9_ aph1Δ* (JJS45) were either treated for 2 h with 20 mM HU or 1 h with 10 µM/ml PL as a positive control. Chk1 activation was visualized by Western blotting showing the band shift of Chk1 to a slower migration form upon activation. **C)** The *aph1Δ* allele in cells containing the partially defective (Myc)_9_‐tagged Cds1 results in higher proliferation in HU (12 mM). Strains *chk1‐HA* (NW222), *chk1‐HA cds1Δ* (JJS43), *chk1‐HA cds1‐(myc)_9_* (JJS44), and *chk1‐HA cds1‐(myc)_9_ aph1Δ* (JJS45) were monitored by growth in a Bioscreen C analyzer. Two independent cultures from the same Bioscreen run are shown per strain and treatment. The curves are representatives of three independent Bioscreen runs. **D)** The (Myc)_9_ tag on Cds1 leads to a compromised function of Cds1 as seen by higher sensitivity against HU but not UV. Logarithmic growing cells of *chk1‐HA* (NW222), *chk1‐HA cds1Δ* (JJS43) *chk1‐HA cds1‐(myc)_9_* (JJS44) *and chk1‐HA cds1‐(myc)_9_ aph1Δ* (JJS45), were serial diluted and spotted on a YES plates as control, a YES plate containing 5 mM HU, or a YES plate placed under UV (200 µJ/cm2).

**Table S1**. *S. pombe* strains used in this study.
