# Supplementary figures and images for: The fission yeast FHIT homolog affects checkpoint control of proliferation and is regulated by mitochondrial electron transport
Source: Cell Biol Int. 2019 Oct 2;44(2):412–23. doi: 10.1002/cbin.11241 (PMC7003880; doi:10.1002/cbin.11241)

A

Fraction PI-stained cells

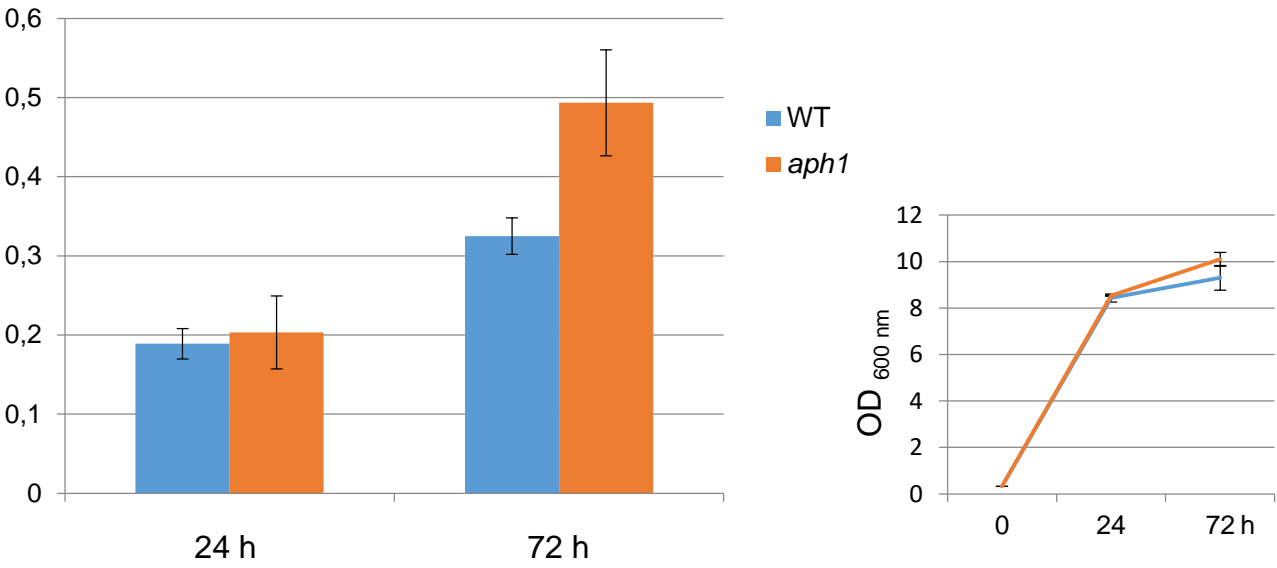

B

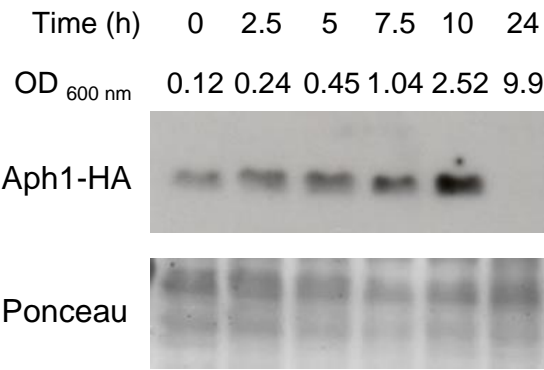

Supplement: Supplementary file 1 — Figure S1. Aph1 is needed for adaptation to stationary phase.A,B) aph1Δ mutants are sensitive to prolonged stationary phase. A) OD600 nm was measured at 24 and 72 h from inoculation. The growth curves show that both wt (972 h‐) and aph1Δ (JJS30) reach the same maximal density at 24 h. B) At 24 h, wt (972 h ‐) and aph1Δ (JJS30) cells are equally effective at excluding PI, whereas at 72 h aph1Δ mutants have more PI permeable cells, indicating that these cells are no longer viable. [file CBIN-44-412-s001.pdf]

**A**

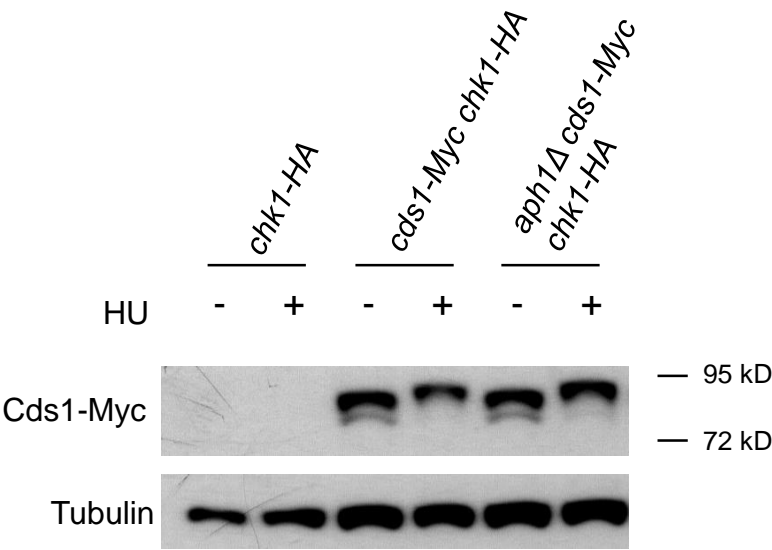

**B**

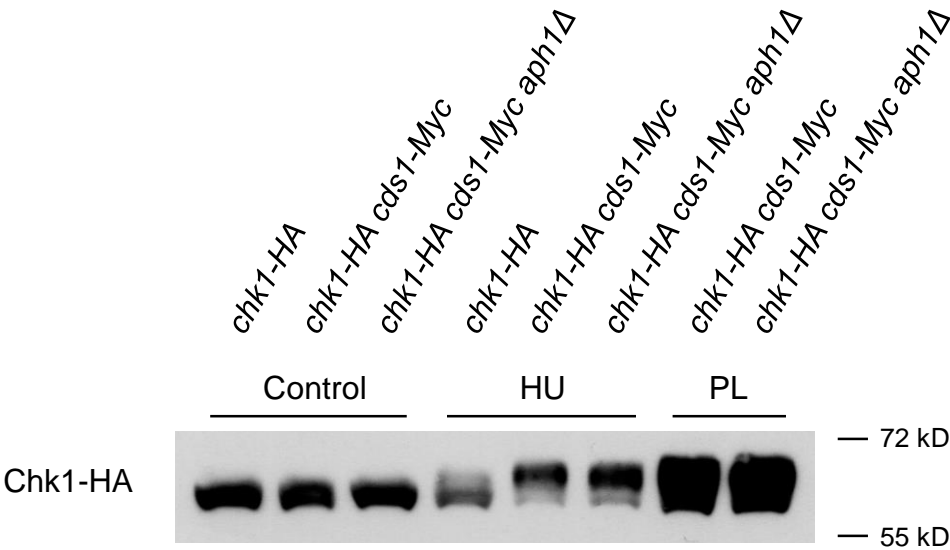

**C**

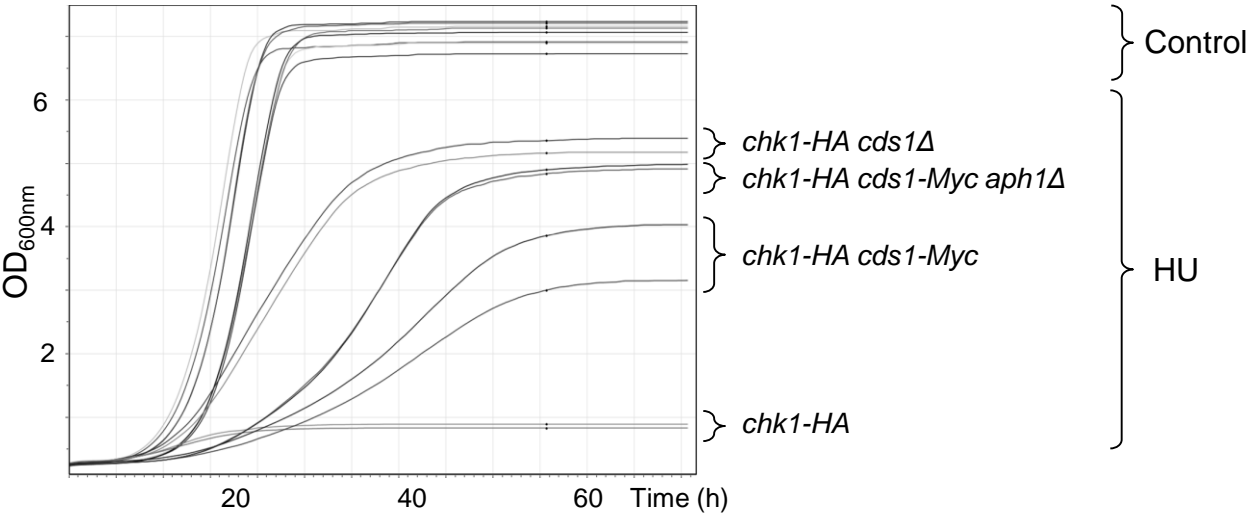

**D**

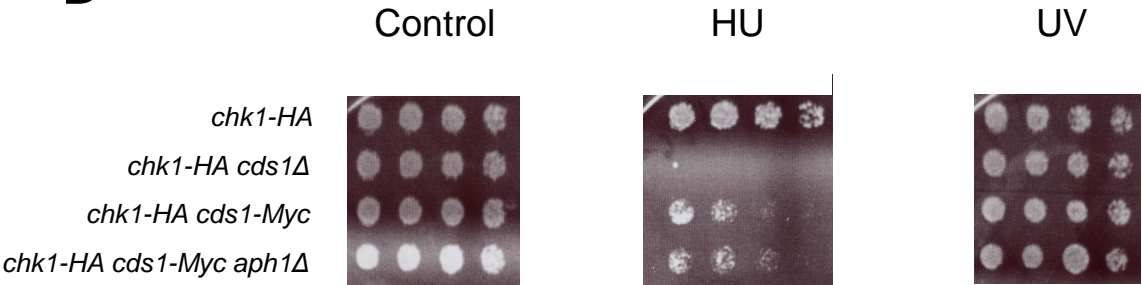

Supplement: Supplementary file 2 — Figure S2. cds1‐(myc)9 is a hypomorphic allele, and aph1Δ in this background results in more proliferation than wt under exposure to DNA damaging agents. A) When activated by HU, Cds1 C‐terminally tagged with (Myc)9 migrates slower as expected, and the aph1Δ allele does not change this. chk1‐HA (NW222), chk1‐HA cds1‐(myc)9 (JJS44) and chk1‐HA cds1‐(myc)9 aph1Δ (JJS45) were treated for 2 h with 20 mM HU, and activation of Cds1 was investigated through Western blotting by presence of a band shift to a slower migrating band. B) The compromised Cds1 function caused by the (Myc)9 tag results in Chk1 activation in HU, indicating DNA damage. Strains chk1‐HA (NW222), chk1‐HA cds1Δ (JJS43), chk1‐HA cds1‐(myc)9 (JJS44), and chk1‐HA cds1‐(myc)9 aph1Δ (JJS45) were either treated for 2 h with 20 mM HU or 1 h with 10 µM/ml PL as a positive control. Chk1 activation was visualized by Western blotting showing the band shift of Chk1 to a slower migration form upon activation. C) The aph1Δ allele in cells containing the partially defective (Myc)9‐tagged Cds1 results in higher proliferation in HU (12 mM). Strains chk1‐HA (NW222), chk1‐HA cds1Δ (JJS43), chk1‐HA cds1‐(myc)9 (JJS44), and chk1‐HA cds1‐(myc)9 aph1Δ (JJS45) were monitored by growth in a Bioscreen C analyzer. Two independent cultures from the same Bioscreen run are shown per strain and treatment. The curves are representatives of three independent Bioscreen runs. D) The (Myc)9 tag on Cds1 leads to a compromised function of Cds1 as seen by higher sensitivity against HU but not UV. Logarithmic growing cells of chk1‐HA (NW222), chk1‐HA cds1Δ (JJS43) chk1‐HA cds1‐(myc)9 (JJS44) and chk1‐HA cds1‐(myc)9 aph1Δ (JJS45), were serial diluted and spotted on a YES plates as control, a YES plate containing 5 mM HU, or a YES plate placed under UV (200 µJ/cm2). [file CBIN-44-412-s002.pdf]
