# Supplementary material for: The fission yeast FHIT homolog affects checkpoint control of proliferation and is regulated by mitochondrial electron transport
Source: Cell Biol Int. 2019 Oct 2;44(2):412–23. doi: 10.1002/cbin.11241 (PMC7003880; doi:10.1002/cbin.11241)
Supplement: Supplementary file 3 — Table S1. S. pombe strains used in this study. [file CBIN-44-412-s003.docx]

Supplementary Fig. S1. Aph1 is needed for adaptation to stationary phase.

A,B) ***aph1Δ* mutants are sensitive to prolonged stationary phase.**

A) **OD_600 nm_ was measured at 24 and 72 h from inoculation. The growth curves show that both wt (972*h^-^*) and *aph1Δ* (JJS30) reach the same maximal density at 24 h.**

B) **At 24 h, wt (972*h^-^*) and *aph1Δ* (JJS30) cells are equally effective at excluding PI, whereas at 72 h *aph1Δ* mutants have more PI permeable cells, indicating that these cells are no longer viable.**

C) **The Aph1 protein level was followed by Western blotting in a strain, JJS31, which expresses a (HA)_3_-tag in the C-terminal of Aph1. The Aph1 level was rising during logarithmic growth, but was absent when reaching maximum density,**

Supplementary Figure S2. *cds1-(myc)_9_* is a hypomorphic allele, and *aph1****Δ*** in this background results in more proliferation than wt under exposure to DNA damaging agents.

A) **When activated by HU, Cds1 C-terminally tagged with (Myc)_9_ migrates slower as expected, and the *aph1Δ* allele does not change this. *chk1-HA* (NW222)*, chk1-HA cds1‑(myc)_9_***  **(JJS44) *and chk1-HA cds1-(myc)_9_***  ***aph1Δ* (JJS45) were treated for 2 h with 20 mM HU, and activation of Cds1 was investigated through Western blotting by presence of a band shift to a slower migrating band.**

B) **The compromised Cds1 function caused by the (Myc)_9_ tag results in Chk1 activation in HU, indicating DNA damage. Strains *chk1-HA* (NW222)*, chk1-HA cds1Δ* (JJS43), *chk1-HA cds1‑(myc)_9_***  **(JJS44), and *chk1-HA cds1‑(myc)_9_***  ***aph1Δ* (JJS45) were either treated for 2 h with 20 mM HU or 1 h with 10 µM/ml PL as a positive control. Chk1 activation was visualized by Western blotting showing the band shift of Chk1 to a slower migration form upon activation.**

C*)* **The** ***aph1Δ* allele in cells containing the partially defective (Myc)_9_-tagged Cds1 results in higher proliferation in HU (12 mM). Strains *chk1‑HA* (NW222), *chk1-HA cds1Δ* (JJS43), *chk1-HA cds1-(myc)_9_***  **(JJS44), and *chk1-HA cds1‑(myc)_9_*** ***aph1Δ* (JJS45) were monitored by growth in a Bioscreen C analyzer. Two independent cultures from the same Bioscreen run are shown per strain and treatment. The curves are representatives of three independent Bioscreen runs.**

D) **The (Myc)_9_ tag on Cds1 leads to a compromised function of Cds1 as seen by higher sensitivity against HU but not UV. Logarithmic growing cells of *chk1-HA* (NW222)*, chk1-HA cds1Δ* (JJS43) *chk1-HA cds1-(myc)_9_* (JJS44) and *chk1‑HA cds1-(myc)_9_***  ***aph1Δ* (JJS45), were serial diluted and spotted on a YES plates as control, a YES plate containing 5 mM HU, or a YES plate placed under UV (200 µJ/cm^2^).**
